# Accuracy Analysis of International Reference Ionosphere 2016 and NeQuick2 in the Antarctic

**DOI:** 10.3390/s21041551

**Published:** 2021-02-23

**Authors:** Zihuai Guo, Yibin Yao, Jian Kong, Gang Chen, Chen Zhou, Qi Zhang, Lulu Shan, Chen Liu

**Affiliations:** 1 School of Geodesy and Geomatics, Wuhan University, Wuhan 430079, China; whugzh@whu.edu.cn (Z.G.); qizhangsgg@whu.edu.cn (Q.Z.); llshan@whu.edu.cn (L.S.); lcahqy@whu.edu.cn (C.L.); 2 Key Laboratory of Geospace Enviroment and Geodesy, Ministry of Education, Wuhan University, Wuhan 430079, China; 3 Collaborative Innovation Center for Geospatial Technology, Wuhan 430079, China; 4 Chinese Antarctic Center of Surveying and Mapping, Wuhan 430079, China; jkong@whu.edu.cn; 5 College of Marine Science and Technology, China University of Geosciences, Wuhan 430074, China; ddwhcg@cug.edu.cn; 6 School of Electronic Information, Wuhan University, Wuhan 430072, China; chenzhou@whu.edu.cn

**Keywords:** International Reference Ionosphere model, NeQuick model, Antarctic, GNSS STEC, accuracy analysis

## Abstract

Global navigation satellite system (GNSS) can provide dual-frequency observation data, which can be used to effectively calculate total electron content (TEC). Numerical studies have utilized GNSS-derived TEC to evaluate the accuracy of ionospheric empirical models, such as the International Reference Ionosphere model (IRI) and the NeQuick model. However, most studies have evaluated vertical TEC rather than slant TEC (STEC), which resulted in the introduction of projection error. Furthermore, since there are few GNSS observation stations available in the Antarctic region and most are concentrated in the Antarctic continent edge, it is difficult to evaluate modeling accuracy within the entire Antarctic range. Considering these problems, in this study, GNSS STEC was calculated using dual-frequency observation data from stations that almost covered the Antarctic continent. By comparison with GNSS STEC, the accuracy of IRI-2016 and NeQuick2 at different latitudes and different solar radiation was evaluated during 2016–2017. The numerical results showed the following. (1) Both IRI-2016 and NeQuick2 underestimated the STEC. Since IRI-2016 utilizes new models to represent the F2-peak height (hmF2) directly, the IRI-2016 STEC is closer to GNSS STEC than NeQuick2. This conclusion was also confirmed by the Constellation Observing System for Meteorology Ionosphere and Climate (COSMIC) occultation data. (2) The differences in STEC of the two models are both normally distributed, and the NeQuick2 STEC is systematically biased as solar radiation increases. (3) The root mean square error (RMSE) of the IRI-2016 STEC is smaller than that of the NeQuick2 model, and the RMSE of the two modeling STEC increases with solar radiation intensity. Since IRI-2016 relies on new hmF2 models, it is more stable than NeQuick2.

## 1. Introduction

The ionosphere is an ionized region with an altitude from 60 km to 2000 km. According to the distribution of ionospheric electron density by height, the ionosphere is generally divided into D (60~90 km), E (90~140 km), F1 (140~210 km), and F2 (over 210 km) layers from low to high. Evaluating total electron content (TEC) is important for characterizing the ionosphere. TEC is measured in electrons per square meter. By convention, 1 TEC Unit (TECu) = $10^{16}$ electrons/$m^{2}$. Owing to the influence of the electron content in the ionosphere, the velocity and path of electromagnetic waves passing through this region were affected to different degrees. Thus, regarding the technique that relies on radio transmission, it is vital to investigate the spatial distribution of ionospheric electrons [1]. To improve the accuracy of satellite navigation and positioning as well as to satisfy the requirements of high-precision measurement, methods for calculating TEC and some ionosphere empirical models, such as the International Reference Ionosphere model and the NeQuick model, have been developed according to the characteristics of the ionosphere and radio transmissions.

The International Reference Ionosphere (IRI) and NeQuick models are two international ionospheric empirical models, which are widely used worldwide [2,3]. The IRI model is based on a large amount of ground-based and space-based observational data, which are mainly from satellites, incoherent scattering radars, and altimeters. The input variables of the IRI model include date, coordinates, solar index, ionospheric index, and magnetic index. The height range of the electron density is 65–2000 km daytime and 80–2000 km nighttime. The model provides ionospheric variables, e.g., monthly average electron density, electron temperature, ion temperature, ion composition, etc. [4]. Thus far, several versions have been released, including IRI-1990, IRI-2000, and IRI-2007. IRI-2016 is the current version. Compared to previous versions, the Altadill et al. The F2-peak height hmF2 (called AMTB from Hereon) model [5] and the satellite and Digisonde model of the F2 layer (SDMF2) model [6] are proposed to estimate the F2-peak height (hmF2) directly in the IRI-2016 model. In addition, the description of the top-level ion composition at lower solar activity is more accurate. The IRI-2016 model can also provide the vertical total electron content (VTEC) from the bottom of the ionosphere to the specified upper boundary. She L.L. et al. [3] compared the F2 layer peak electron density (NmF2) and hmF2 data from IRI-2016 to ionosonde data during geomagnetic quiet periods to estimate the model’s accuracy and precision. The results indicate that IRI predictions were in good agreement with ionosonde data for both NmF2 and hmF2, and IRI predictions were systematically higher than the ionosonde observed results.

The NeQuick model is a time-dependent, three-dimensional ionospheric electron density model [7]. Based on hmF2, the model is divided into bottom and top models. The input variables are height, geocentric latitude, geocentric longitude, solar activity (given by monthly mean sunspot number R12 or 10.7 cm solar radio flux F10.7), and Universal Time (UT). The NeQuick provides services to evaluate the electron density along any ground-to-satellite, straight-line ray path and the corresponding TEC by numerical integration [7]. The NeQuick model includes two versions of NeQuick1 and NeQuick2 [8]. Compared to NeQuick1, the modified analytical model of the electron density profile originally proposed by G. Di Giovanni and S. M. Radicella (DGR model) [9,10] is used in NeQuick2 to describe the ionospheric electron density from 90 km upper to the F2 layer peak. Three profile anchor points (the E-layer peak, the F1 layer peak, and the F2 layer peak) are modeled in terms of the inonsonde parameters contains the E-peak plasma frequency (foE), the F1-peak plasma frequency (foF1), the F2-peak plasma frequency (foF2), and M(3000)F2 [2]. Wang et al. [11] evaluated the accuracy of global positioning system (GPS) broadcast Klobuchar, original NeQuick2, and fitted NeQuickC as well as Galileo broadcast NeQuickG models over the continental and oceanic regions, respectively. The results showed that NeQuickG can mitigate ionospheric delay by 54.2–65.8% on a global scale and NeQuickC can correct for 71.1–74.2% of the ionospheric delay. NeQuick2 performs at the same level as NeQuickG, which is slightly better than that of the GPS broadcast Klobuchar model. Angrisano et al. [4] compared the ionospheric delay error of the NeQuick and the Klobuchar model with reference computed using final global ionospheric maps (GIMs). The results indicated that the accuracy of the NeQuick model was double that of the Klobuchar model.

To compare and demonstrate the modeling accuracy of IRI and NeQuick, researchers have performed many studies. Cherniak et al. [12] analyzed the modeling accuracy of NeQuick2 and plasmasphere extension of the IRI model (IRI-Plas) during periods of quiet and medium solar activity on a global scale. It was shown that both IRI-Plas and NeQuick2 overestimated the electron content in the 250–500-km altitude interval for low solar activity and the IRI-Plas model overestimated TEC for the 500–20,000-km altitude range during daytime local time at low and moderate solar activities, while NeQuick2 underestimated values for all considered seasons and local time. Venkatesh et al. [13] analyzed the accuracy of the IRI-2012 model and the NeQuick model in equatorial and low-latitude areas and found that the strength and the locations of the equatorial ionization anomaly (EIA) crest are nearly well represented by both models during low solar activity, while the models underestimate the peak TEC at the EIA during the increased solar activity conditions. The evaluated results of anomaly equatorial ionizations in both models are also lower than the true values during the high solar activity period. Fang et al. [14] compared the modeling accuracy of the IRI model, NeQuick model, and Klobuchar model in low-latitude and mid- to high-latitude areas. The results showed that the NeQuick model and the IRI-2007 model could better evaluate the bimodal structure on both sides of the equator during the daytime. However, the exact value and position of the peak could not be obtained, and the accuracy of the two models in the mid- or high-latitude areas was higher than in low-latitude areas. Jun C. et al. [15] assessed the accuracy and reliability of the International Global Navigation Satellite System Global Ionospheric Maps (IGSG), NeQuick2, and IRI-2016 by applying different assessment methods. The results showed that, in both precision index statistics and positioning applications, the performance of IGSG is better than that of NeQuick2 and IRI-2016. Daniel Okoh et al. [16] investigated the climatological assessment of the NeQuick and IRI-Plas models at a global scale using global navigation satellite system (GNSS) observations. The results showed that both IRI-Plas and NeQuick were fairly accurate in trends with the GNSS measurements. The NeQuick predictions were generally better than IRI-Plas predictions for most stations and times. Due to the lack of observational data, especially near the South Pole, relevant studies have mainly concentrated on mid- and lower-latitude regions and few in the South region. In addition, no relevant studies have used data from the South Pole. Yekoye A.T. [17] focused on the pattern of variation of the VTEC inferred from the GPS, IRI 2016, IRI-Plas 2017, and NeQuick 2 with different heights over Antarctica. The results showed that the GPS-derived (GPS VTEC) and -modeled (IRI 2016, IRI-Plas 2017 and NeQuick 2) VTEC did not show significant variations in the hourly values. The modeling accuracy performance of NeQuick2 was better than that of IRI-Plas 2017. With improvements in science, technology, and logistics support capabilities in several countries, the scope of Antarctic scientific expedition is gradually expanding toward the South Pole. As such, a reliable ionospheric empirical model is vital for reliable navigation and positioning for Antarctic scientific expeditions. Based on the abovementioned factors, in this study, the observational data from five Antarctic International GNSS Service (IGS) stations [18], six Polar Earth Observing Network (POLENET) stations, and some GNSS stations near the South Pole are used to calculate the GNSS STEC of the Antarctic. By comparing the GNSS STEC with the STEC evaluated by the IRI-2016 and NeQuick2 models, this study statistically analyzed and compared the accuracy of the two models.

## 2. Data and Methodology

### 2.1. Data Description

**Figure 1:**
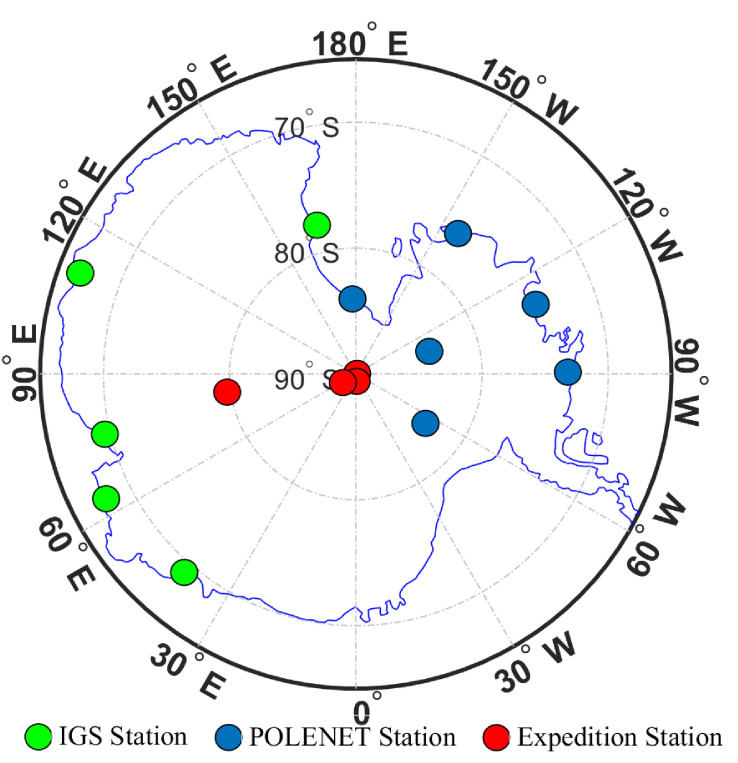
Distribution diagram of Global Navigation Satellite System stations in Antarctica.

The observational data used in this study were the dual-frequency observational data from GNSS stations in the Antarctic. These stations include five IGS stations, six Polar Earth Observing Network (POLENET) stations, and four GNSS stations built by members of the Antarctic expedition. The distribution of all these stations is shown in Figure 1. The observational data were collected in December 2016, March 2017, June 2017, and September 2017, while the data from four GNSS stations in December 2016 were collected by Antarctic expedition members.

### 2.2. GNSS STEC

Because the ionospheric delay of GNSS signals is inversely proportional to the square of the signal frequency, GNSS dual-frequency observation data can be used to calculate the STEC of the signal propagation path [19]. As the pseudo-range measurement noise is higher than the carrier phase measurement noise, the method of dual-frequency carrier phase smoothing pseudo-ranges can be used to calculate the STEC value with high precision. The *TEC* calculation formula for the carrier phase smoothing pseudo-ranges is shown in Equation (1) [20,21].

(1) $$TEC=\frac{f_{1}^{2}f_{2}^{2}}{40.28(f_{1}^{2}-f_{2}^{2})}[L_{4}-{\langle P_{4}+L_{4}\rangle }_{arc}+(c\cdot B_{r}+c\cdot B^{s})]$$

$L_{4}$ and $P_{4}$ are the geometric-free combination observations formed between frequency $f_{1}$ and $f_{2}$ by pseudo-range and carrier phase observations, respectively; 〈 〉${}_{arc}$ represents the average value within a continuous arc; c means the propagation speed of rays in vacuum; and $B_{r}$ and $B^{s}$ represent the differential code bias (DCB) of the receiver and satellite, respectively.

In this study, the aforementioned method was used to calculate ionospheric GNSS STEC in the Antarctic. To ensure the accuracy of the calculation results, the cutoff of the satellite elevation angle was set as 15°. From Equation (1), it can be concluded that the accuracy of $B_{r}$ and $B^{s}$ directly affects the TEC calculation results. Thus, the second-order polynomial surface fitting model was used to estimate the DCBs of receivers and satellites as follows:

(2) $$\sum_{i=0}^{n}\sum_{j=0}^{m}E_{ij}{\left(\varphi -\varphi_{0}\right)}^{i}{\left(S-S_{0}\right)}^{j}=TEC\cdot \cos\chi$$

$E_{ij}$ is the coefficient to be evaluated of the polynomial model and m and *n* are the orders of the polynomial. In this equation, the polynomial model is in order to 3×2. The $\varphi_{0}$ is the geographic latitude of the center of measurement area, $S_{0}$ is the solar hour angle of the measurement area center at the central moment $t_{0}$ of this period, and $S-S_{0}=\left(\lambda -\lambda_{0}\right)+\left(t-t_{0}\right)$ yields the difference of the solar hour angle, where $\lambda_{0}$ is the geographic longitude of the measurement area center, λ is the geographic longitude of the Ionospheric Puncture Point (IPP), the height of which is about 350 km, t is the epoch of observation, and χ is the zenith angle of satellite. Through Equations (2) and (3), the ionospheric model equation can be obtained as follows:

(3) $$\overset{n}{\underset{i=0}{\sum }}\overset{m}{\underset{j=0}{\sum }}E_{ij}{\left(\varphi -\varphi_{0}\right)}^{i}{\left(S-S_{0}\right)}^{j}-\frac{c\cdot f_{1}^{2}f_{2}^{2}\cdot cos\chi }{40.28(f_{1}^{2}-f_{2}^{2})}\left(B_{r}+B^{S}\right)=\frac{f_{1}^{2}f_{2}^{2}\cdot cos\chi }{40.28(f_{1}^{2}-f_{2}^{2})}\left[L_{4}-P_{4}+L_{4}{}_{arc}\right]$$

Equation (3) indicates that DCBs of receivers and satellites and polynomial coefficients $E_{ij}$ together constitute unknown parameters for least square solution. In this condition, the coefficient matrix of the normal equation was rank deficient. Therefore, the following constraint conditions were adopted in this paper [22]:

(4) $$\overset{m}{\underset{i=0}{\sum }}q^{i}=0$$

where m means the number of satellites and $q^{i}$ is the DCB of satellite *i*. According to the least square principle, using $E_{ij}$, the DCBs of satellites and receivers can be calculated. Polynomial surface fitting model is a widely used ionospheric model. Many studies have shown that the model is suitable for regional ionospheric modeling and can achieve high accuracy, which was suitable for the study in this paper.

### 2.3. IRI-2016 STEC

**Figure 2:**
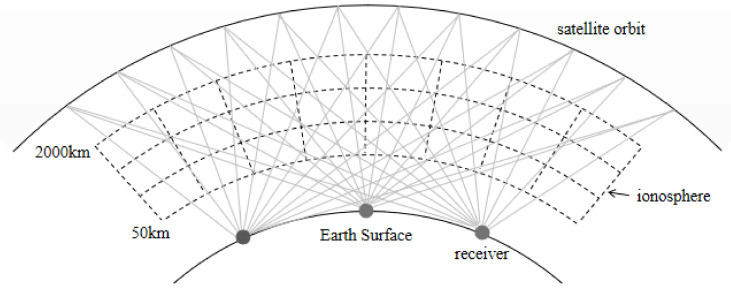
Schematic diagram of the spatial grid model.

For the IRI-2016 model, the vertical electron density of the top side (top of hmF2) and bottom side (bottom of hmF2) could be calculated using different formulations. The information of vertical electron density is described in Table 1 in detail. The electron density at any point in the ionospheric region can be calculated by interpolation. Then, the obtained electron density can be used to compute STEC values via integration in the GNSS signal propagation path, as shown in Figure 2 and Equation (5).

(5) $$STEC=\int_{l}Ne(r,t)ds$$

*STEC* is the slant TEC of the ionosphere evaluated by the IRI-2016 model, *l* is the radio wave propagation path, and $Ne\left(r,t\right)$ is the electron density in the direction r at time t.

### 2.4. NeQuick2 STEC

Based on the Consultative Committee on International Radio (CCIR) files, NeQuick2 uses specific parameters such as the electron density peak parameter of the F2 layer (NmF2), F2 peak height (hmF2), F2 layer spreading factor (M(3000)F2), and the solar activity index (F10.7) to calculate the electron density in the ionosphere at a certain time. Then, by integrating the electron density, the STEC can be evaluated by the NeQuick2 mode. Nava et al. [23] presented the calculation method hmF2 or STEC using the NeQuick model in detail. 

### 2.5. COSMIC Electron Density Profile

The Constellation Observing System for Meteorology Ionosphere and Climate (COSMIC) is a satellite mission for the Earth sciences to solve some of the most important geo-scientific issues today. Placing a constellation of GPS receivers in low-Earth orbit, COSMIC provides an extremely powerful system for continuously and extensively measuring the ionosphere [24]. COSMIC, using GPS occultations, can provide the electron density profile at the height of the E and F layers of the ionosphere, where the standard deviation compared with digital altimeter results is less than 20% [25,26].

## 3. Data Processing and Analysis

### 3.1. Reliability Analysis of GNSS STEC

The accuracy of GNSS STEC depends on the accuracy of the DCBs of receivers and satellites. Thus, the DCBs estimated in this study were compared with DCBs provided by the Center for Orbit Determination in Europe (CODE). The precision of the estimated DCBs of 32 satellites and DCBs of five IGS stations were evaluated by analyzing the statistics bias and STD, as shown in Table 2 and Table 3.

As seen from Table 2, compared with DCBs of satellites provided by CODE, the mean bias and mean standard deviation (STD) of estimated DCBs were, respectively, 0.35 ns and 0.13 ns. From Table 3, the mean bias and mean STD of the five IGS stations (CASI, DAV1, MAW1, MCM4, SYOG) were 0.193 ns, 0.276 ns, 0.129 ns, 0.401 ns, and 0.221 ns and 0.091 ns, 0.137 ns, 0.075 ns, 0.125 ns, and 0.069 ns, respectively. The result indicates that the estimated result of DCB is reliable and consistent with the DCB of CODE. According to the conclusions of the study [21] and existing experimental results, the calculated GNSS STEC can be considered as the true STEC.

### 3.2. Comparison of Different Models

In contrast to previous research methods, we compared modeling *STEC* and *GNSS STEC* rather than VTEC to avoid errors introduced in the projection process. Based on the analysis presented above, the calculated *GNSS STEC* can be regarded as the true value of *STEC*. To investigate the accuracy of the *IRI-2016 STEC* and *NeQuick2 STEC*, in this study, the difference and its absolute value between the *GNSS STEC* and the modeling *STEC* (*IRI-2016 STEC* or *NeQuick2 STEC*) were computed, as shown in Equation (6).

(6) $$dSTEC_{IRI-2016}=STEC_{IRI-2016}-STEC_{GNSS}\;dSTEC_{NeQuick2}=STEC_{NeQuick2}-STEC_{GNSS}$$

### 3.3. Influence of Solar Radiation

In high-latitude areas, the ionosphere dynamic depends on not only the background solar radiation, but also on the electron and proton fluxes and, especially, on their variation geomagnetic storms. The disturbance storm time (Dst) index [27] is obtained by measuring hourly changes in geomagnetic horizontal component intensity to monitor global geomagnetic activity and equatorial circulation intensity. The change in the Dst index is generally used to characterize each stage of the storm and to distinguish the intensity of the storm. When a magnetic storm occurs (Dst index higher than −30 nT), the Dst index values will decrease, and the smaller the Dst index, the stronger the storm. The change in the Dst index during the experimental period was investigated and the results showed that it was all higher than −10 nT. According to the existing research results, it could be concluded that no geomagnetic storm occurred during the experiment and the experimental results were little affected by geomagnetic activity.

In the Antarctic, the solar radiation intensity is different in different months and is also different at different latitudes within the same month. Since the ionospheric electron density and TEC are both greatly influenced by solar radiation, it is crucial to consider the influence of solar radiation when assessing the accuracy of the empirical model of the Antarctic area. In June, the Antarctic is in Polar Night and almost all regions are not affected by solar radiation. In December, it is Polar Day and almost all regions are directly exposed to solar radiation. In March and September, half of the Antarctic area is on the day side and the other half is night side. Solar radiation on the day side gradually decreases as latitude increases, while the night side is more affected by solar radiation in high-latitude areas. 

**Figure 3:**
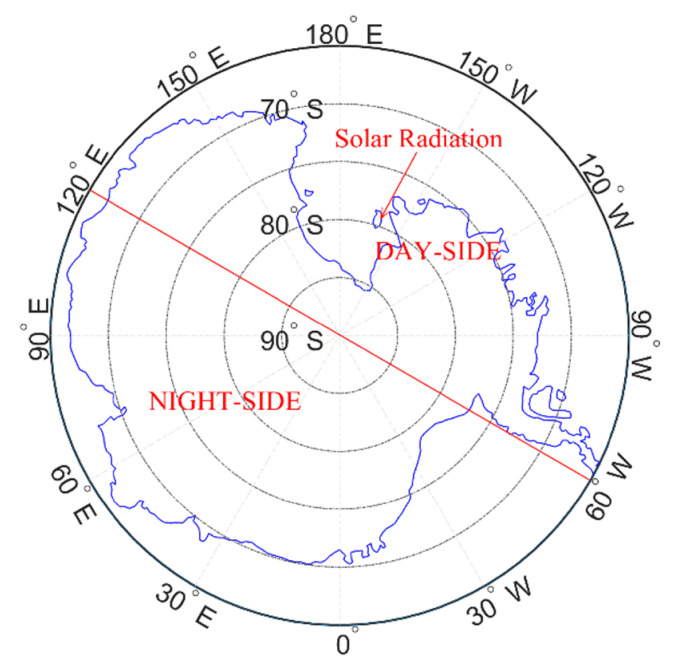
Division of latitudes in the illumination area and back-light area of the Antarctica at 02:00 UT during March and September.

Since the radiation conditions in March and September are basically the same, the Antarctic is divided into day side and night side (at 2:00 UT, the day side contains an area of 120° E to 180° and 60° W to 180° and the night side is 0° to 120° E and 0° to 60° W. At 14:00 Universal Time, the opposite occurs.). Then, both the day side and the night side are divided into six latitude bands with intervals of 5° in latitude, as shown in Figure 3. The bias and root mean square (RMS) values were counted to compare the accuracy of the two models of STEC in different latitude bands with different solar radiation. Similarly, in June and December, the Antarctic was divided into six latitude bands (without day side and night side), and bias and RMS in each band were separately counted.

### 3.4. Comparison of COSMIC and Models

In this paper, the experimental evaluation results were assessed by the statistics of TEC deviations provided by *IRI-2016*, *NeQuick2*, and *COSMIC* in different layers. *COSMIC* data, whose geographic coordinates are within the Antarctic (60° S to 90° S) during corresponding periods, were chosen. In addition, *COSMIC* data with an incomplete or questionable electron density (Ne) profile were rejected. Rate of misestimation of models in each ionospheric layer were calculated, as shown below:

(7) $$RME_{model}^{L}=\frac{1}{N}\sum_{i}^{N}\frac{TEC_{model,i}^{L}-TEC_{COSMIC,i}^{L}}{TEC_{cosmic,i}^{L}}$$

*RME* means the rate of misestimation, *L* represents different layers (E, F1, and F2) of ionosphere, model means *IRI-2016* or *NeQuick2*, and *N* is total number of occultation observations.

## 4. Results

### 4.1. Comparison of Modeling STEC

**Figure 4:**
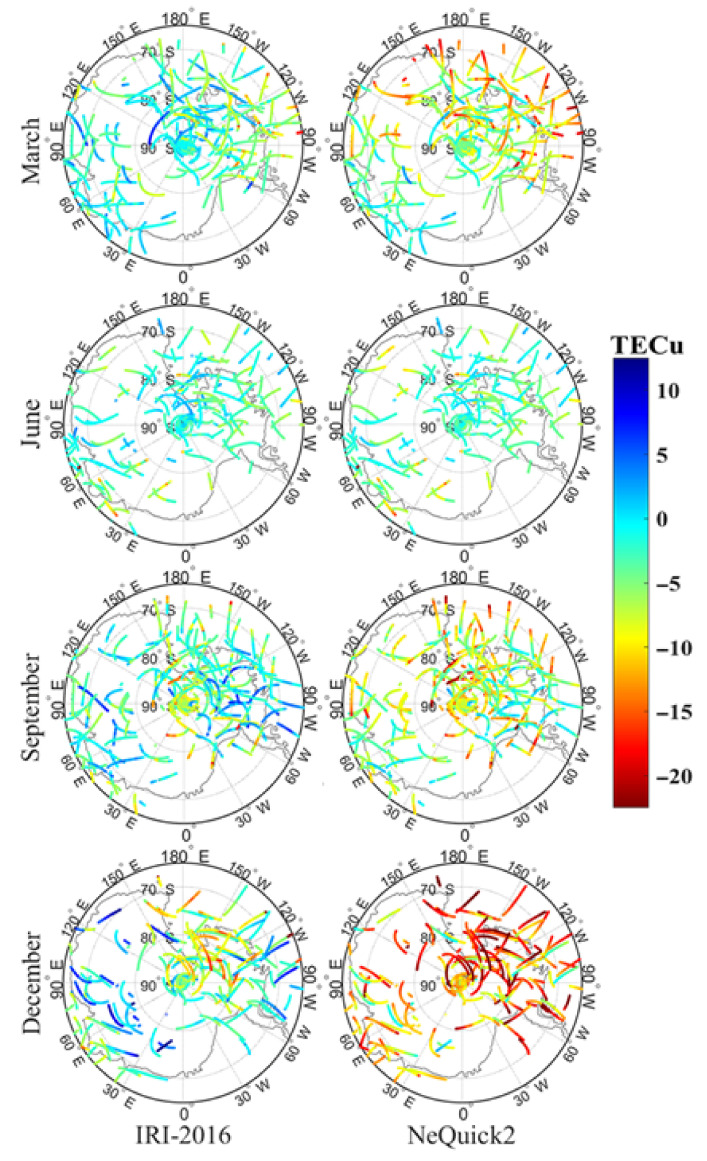
Distribution of difference of STEC (dSTEC) of the International Reference Ionosphere-2016 and NeQuick2 models at 2:00 UT during the experiment period.

**Figure 5:**
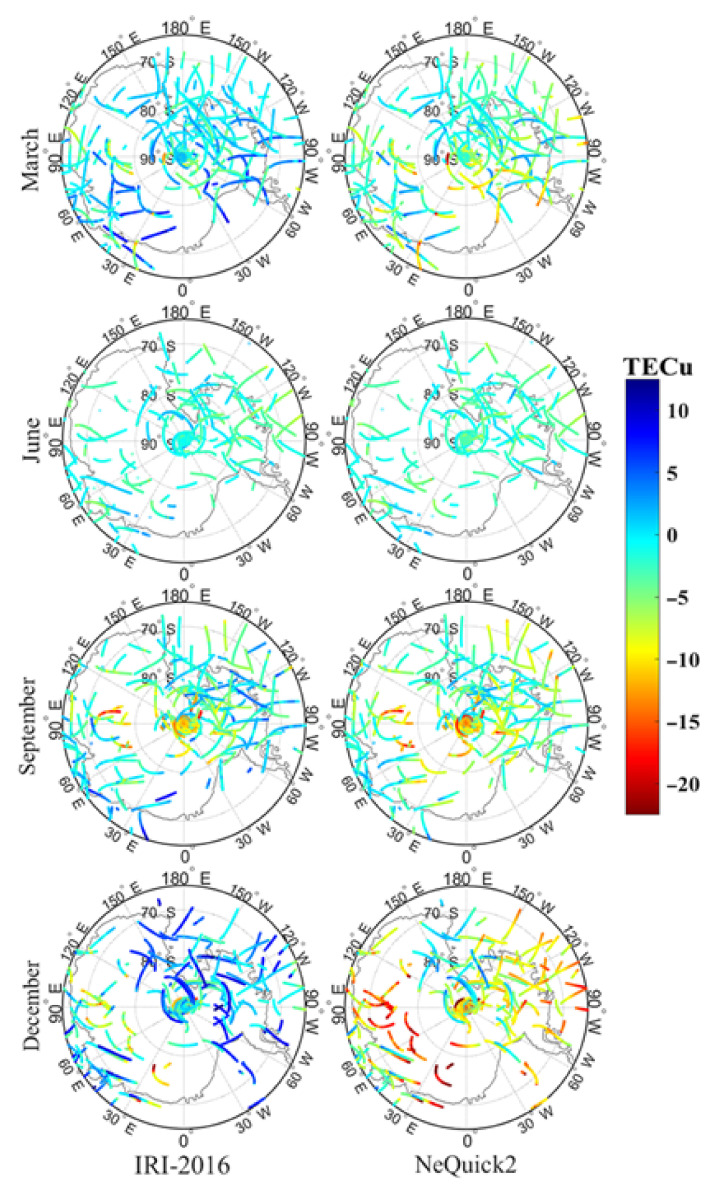
Distribution of difference of STEC (dSTEC) of the International Reference Ionosphere-2016 and NeQuick2 models at 14:00 UT during the experiment period.

**Figure 6:**
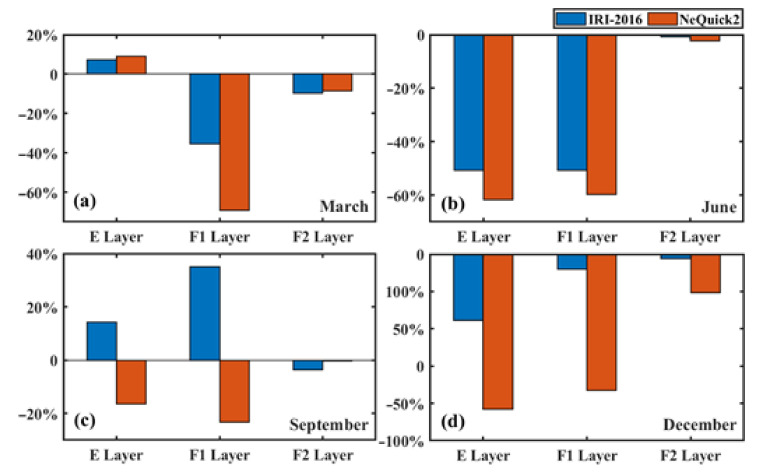
Statistics on the rate of misestimation of models in each ionosphere layers. E, F1, and F2 layers represent the altitude range of 100–140 km, 140–210 km, and 210–800 km, respectively. Figures (**a**–**d**) mean statistical results in March, June, September, and December, respectively.

The dSTEC values of the two models with GNSS STEC are shown in Figure 4 and Figure 5, corresponding to 02:00 UT and 14:00 UT in the four experimental months. In these two figures, the projection height is 300 km (i.e., the ionospheric electron density peak height). Based on the analysis of the projection, it is apparent that the IRI-2016 STEC value was higher than the NeQuick2 value and the difference varied with time. In June, the dSTEC of the two models showed little difference. It was basically maintained within 2 TECu, and the two modeling STECs were basically the same. In March and September, the difference for the NeQuick2 model was significantly higher than that of the IRI-2016 model, and the IRI-2016 STEC was approximately 3–10 TECu higher than NeQuick2 STEC, but the difference between the two models was basically consistent in March and September. In December, the difference was further expanded. As shown in Figure 4 and Figure 5, the IRI-2016 STEC in December was about 15–20 TECu higher than NeQuick2 STEC. By comparing the results with COSMIC occultation data, it was concluded, from Figure 6, that at the E, F1, and F2 layers, the IRI-2016 misestimated TEC by about 7.23%, −35.41%, and −9.64% in March, −50.78%, −23.2%, and −0.87% in June, 14.22%, 35.13%, and −3.68% in September, and −17.75%, −4.08%, and −1.13% in December, respectively; NeQuick2 misestimated about 9%, −69.03%, and −8.39% in March, −61.8%, −59.7%, −2.26% in June, −16.35%, −23.35%, and −0.41% in September, and −41.54%, −36.58%, and −10.38% in December, respectively. Considering the dominance of the electron content in the F2 layer, these experimental results indicated that both models underestimated STEC and that the performance of the NeQuick2 model was more obvious. Different from NeQuick2, which relied on the CCIR model for M(3000)F2 and on the close correlation between hmF2 and M(3000)F2, IRI-2016 used AMTB and SDMF2 models to represent hmF2 directly. This effectively avoided the error source connected with the M(3000)F2 approach and explained that the IRI-2016 STEC was closer to GNSS STEC than NeQuick2. In this study, GNSS STEC was calculated up to 20,200 km and contained not only the ionospheric total electron content (ITEC) but also the plasmaspheric total electron content (PTEC) in the plasma layer, but these two models only calculated the STEC from the receiver to a height of 2000 km. Thus, in addition to the limitations of the accuracy of these two models, PTEC in the plasma layer over the ionosphere was also one of the factors that caused the above results. The global distribution of the plasma layer PTEC was analyzed in detail, and the content of PTEC in the Antarctic was approximately 2 TECu [28], which is significantly less than the difference obtained in this paper. This indicates that the difference in this study could not be completely caused by PTEC.

**Figure 7:**
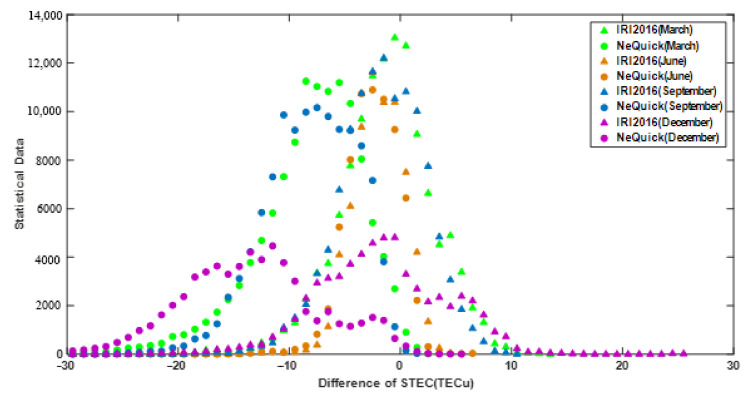
Distribution interval statistics of dSTEC of the IRI-2016 and NeQuick2 models.

To analyze the deviation between the two models’ STEC and GNSS STEC, the difference in STEC was statistically distributed according to a statistical interval of every 1 TECu, as shown in Figure 7. It is apparent that the dSTECs of the two models generally had normal distributions. For the IRI-2016 model, dSTEC obeyed a normal distribution with a zero axis of symmetry. In the case of the 95% confidence level, the confidence interval of dSTEC in June, March, September, and December were (−8, 3), (−14, 10), (−14, 9), and (−18, 12), respectively. The symmetry axis of NeQuick2 model’s normal distribution curve varied with time, which were −2 TECu, −8 TECu, −8 TECu, and −12 TECu in June, March, September, and December, respectively. At a 95% confidence level, the relevant confidence intervals in June, March, September, and December were (−8, 3), (−25, 2), (−23, 2), and (−30, 3). Since the solar radiation intensity in the Antarctic increased successively in June, March, September, and December, it was concluded that the two modeling STECs were both affected by the intensity of solar radiation. Specifically, as solar radiation increased, both the dSTEC and modeling accuracy of the IRI-2016 model and the NeQuick2 model decreased. In addition, the NeQuick2 STEC was underestimated and systematically biased as solar radiation increased. The performance of the IRI-2016 model was less affected by changes in solar radiation and the dSTEC varied little in different seasons. Thus, the IRI-2016 model was more stable. In contrast, the deviation of the NeQuick2 model fluctuated greatly as seasons changed, and the model was more sensitive to changes in solar illumination, which can be considered in the next generation model update.

### 4.2. Influence of Solar Radiation on Modeling Accuracy

**Figure 8:**
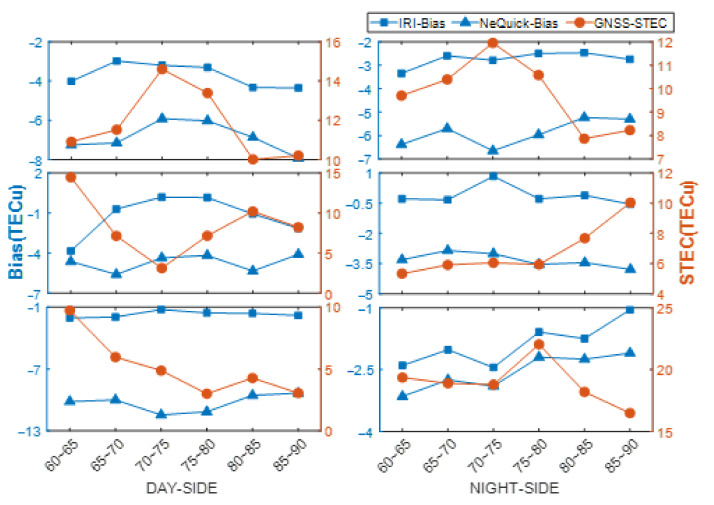
Variation in GNSS STEC and modeling bias values. Three rows are aligned vertically at both left- and right-hand sides, respectively. The left-hand side represents the statistics of the variation of GNSS STEC and bias of two models with latitude in day side, while right-hand side represents in night side. The first row represents GNSS STEC and bias variation of two models from 1:00 to 3:00 in March and September. The second row represents STEC and bias variation of two models from 13:00 to 15:00 in March and September. The third row represents STEC and bias variation of two models in June and December. Day side means polar day and night side means polar night.

**Figure 9:**
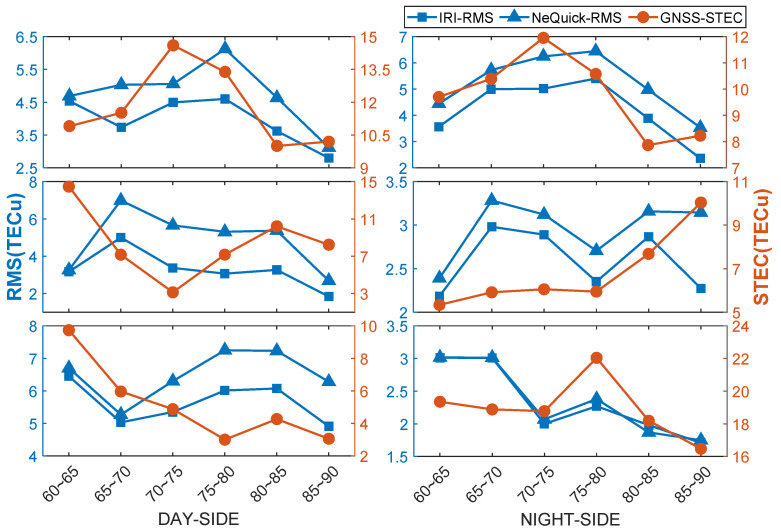
Variation in GNSS STEC and modeling Root Mean Square (RMS) values. Three rows are aligned vertically at both left- and right-hand sides, respectively. Left-hand side represents the statistics of the variation of GNSS STEC and RMS values of the two models with latitude in day side, while right-hand side represents in night side. The first row represents GNSS STEC and RMS variation of two models from 1:00 to 3:00 in March and September. The second row represents STEC and RMS variation of two models from 13:00 to 15:00 in March and September. The third row represents GNSS STEC and RMS variation of two models in June and December. Day side means polar day and night side means polar night.

As seen in Figure 8 and Figure 9, the bias and RMS values of IRI-2016 were both lower than those of NeQuick2. In March and September, the bias of IRI-2016 was about 3 TECu lower than that of NeQuick2 in each latitude band of both day and night sides. On the day side, the bias of both models varied noticeably at different latitudes; that is, the bias in intermediate latitude bands (70–80 degrees S) was lower than that in marginal bands (60–70 degrees S and 80–90 degrees S) and the difference was about 1 TECu. In addition, the bias values of the day side and night side in the intermediate latitude bands were closer than those in the marginal bands. On the night side, the bias of both IRI-2016 and NeQuick2 varied little in different bands without an obvious pattern. The bias values of IRI-2016 and NeQuick2 at 2:00 UT were −3 TECu and −6 TECu, respectively, and 0 TECu and −3 TECu at 14:00 UT. Moreover, the bias of both models on the day side was always higher than that on the night side. As GNSS STEC in the Antarctic is generally small and gradually decreases as latitude increases, within 30 TECu in lower latitudes (60–70 degrees S) and within 15 TECu in higher latitudes (80–90 degrees S), the corresponding accuracy gradually improves, which may be one of the reasons for the phenomenon described above. However, because fewer observation data are available for Antarctica inland, the modeling accuracy near the pole is poor and the bias increases further. The RMS value of IRI-2016 was approximately 0 to 2 TECu lower than that of NeQuick2 on both day and night sides, which is not regular with the change in latitude and solar radiation intensity.

In June and December, the bias values of the two models barely varied with the change in latitude, both in the polar day and polar night. The bias values of the IRI-2016 model during the polar day and polar night were approximately 2 TECu, while those of the NeQuick2 model were quite different. The bias value in each latitude band was approximately 10 TECu during the polar day, while it was approximately 3 TECu during the polar night. The RMS values of the two models were close to each other during the polar night, approximately 2–3 TECu, which were much lower than those during the polar day. The RMS of the NeQuick2 model on a polar day was greater than that of the IRI-2016 model, and the difference increased with latitude, which was nearly 0.1 TECu in lower latitude bands while approximately 1 TECu in higher latitude bands. Accordingly, it was concluded that the stabilities of the two models were both affected by solar radiation. The stabilities of the two models were better on the night side than on the day side, and the stability of the NeQuick2 model was weaker than that of the IRI-2016 model.

The above results show that the performance of the IRI-2016 model was better than that of the NeQuick2 model in terms of the accuracy and stability of the estimation results, and the quality of the two models’ results were both affected by solar radiation. The IRI-2016 model was less affected by solar radiation than the NeQuick2 model. The estimation result of the NeQuick2 model was less stable than the IRI-2016 model on both the day and night sides.

## 5. Discussion and Conclusions

In this study, the GNSS STEC with high accuracy was regarded as the true STEC value by evaluating the accuracy of DCB. The IRI-2016 STEC and NeQuick2 STEC were estimated within the altitude height range of 60 km to 2000 km, respectively. Afterward, the Antarctic was divided into different latitude bands, and the solar radiation difference was also considered in this paper. Based on the above design, the accuracy and stability of the two models were compared by analyzing the difference between the two modeled STEC and the GNSS STEC (dSTEC), bias, and RMS value. The experimental conclusions were as follows:

1.  Both IRI-2016 and NeQuick2 underestimated STEC in the ionosphere in the Antarctic. This may be because the GNSS STEC contains both the ITEC and the PTEC, while the modeled STEC was only estimated up to a height of 2000 km. However, the content of PTEC in the Antarctic is very low and cannot be the only cause of this result. Compared to the two empirical models, the IRI-2016 STEC was closer to the true STEC. In June, the STEC difference between the two models was basically maintained within 2 TECu. In March and September, the NeQuick2 STEC was approximately 3–10 TECu lower than that of the IRI-2016 model. In December, the difference between the two models was more pronounced. Since the introduction of the AMTB and SDMF2 models to simulate hmF2 directly, the IRI-2016 STEC performed significantly better than the NeQuick2 STEC.
2.  Bias of both models did not vary greatly with latitude on the night side but decreased and then increased as latitude increased on the day side. This could be because the GNSS STEC in the Antarctic is generally small and gradually decreases as latitude increases, but the modeling accuracy near the pole was poor owing to the lack of observational data. In the intermediate latitude bands (70–80° S) of the Antarctic, the bias value between day and night side was close, with a difference of approximately 0–1.5 TECu. In the lower (60–70 degrees S) and higher latitudes (80–90° S), the bias value on the day side was larger than that on the night side, with a difference of approximately 1–3 TECu. In addition, the variation of the bias value of the NeQuick2 model between different latitudes was more obvious. In conclusion, the modeling accuracy near the pole must be improved, which can be considered in the next versions of the two models.
3.  The dSTEC values of the two models were both normally distributed. The distribution range of both the modeled dSTEC increased as solar radiation increased, and the NeQuick2 STEC was systematically biased as solar radiation increased.
4.  The RMS value of the IRI-2016 model was always smaller than that of the NeQuick2 model, and the RMS values of the two models increased with solar radiation intensity.

All these results indicate that the estimated results of the two models were more stable under weak solar radiation. Since the IRI-2016 uses new hmF2 models to avoid the error source connected with the M(3000)F2 approach, IRI-2016 is less affected by changes in solar radiation and its performance is more stable.

## Figures and Tables

**Table 1:** Vertical electron density data.

	Latitude (°)	Longitude (°)	Altitude (km)
Range	−60~−90	−180~180	50~2000
Resolution	1	1	50

**Table 2:** Precision statistics of the differential code bias (DCBs) for satellites.

Bias (ns)			STD (ns)		
Mean	Min	Max	Mean	Min	Max
0.35	0.09	0.61	0.13	0.06	0.22

**Table 3:** Precision statistics of DCBs for the International GNSS Service (IGS) stations.

	CAS1	DAV1	MAW1	MCM4	SYOG
Bias (ns)	0.193	0.276	0.129	0.401	0.221
STD (ns)	0.091	0.137	0.075	0.125	0.069

## Data Availability

Not applicable.
